# Essential Oils Modulate Gene Expression and Ochratoxin A Production in *Aspergillus carbonarius*

**DOI:** 10.3390/toxins8080242

**Published:** 2016-08-19

**Authors:** Rachelle El Khoury, Ali Atoui, Carol Verheecke, Richard Maroun, Andre El Khoury, Florence Mathieu

**Affiliations:** 1 Laboratoire de Mycologie et Sécurité Alimentaire (LMSA), Centre d’analyse et de Recherche (CAR), Campus des Sciences et Technologie, Université Saint-Joseph, Mkalles-Beyrouth 1107-2050, Lebanon; rachelle.khouryel@net.usj.edu.lb (R.E.K.); richard.maroun@usj.edu.lb (R.M.); andre.khoury@usj.edu.lb (A.E.K.); 2 Laboratoire de Génie Chimique, Université de Toulouse, CNRS, INPT, UPS, Toulouse 31326, France; carol.verheecke@gmail.com (C.V.); florence.mathieu@ensat.fr (F.M.); 3 Laboratory of Microbiology, Department of Natural Sciences and Earth, Faculty of Sciences I, Lebanese University, Hadath Campus, Beirut P.O Box 11-8281, Lebanon

**Keywords:** *Aspergillus carbonarius*, Ochratoxin A, gene expression, essential oils

## Abstract

Ochratoxin A (OTA) is a mycotoxin, mainly produced on grapes by *Aspergillus carbonarius*, that causes massive health problems for humans. This study aims to reduce the occurrence of OTA by using the ten following essential oils (E.Os): fennel, cardamom, anise, chamomile, celery, cinnamon, thyme, taramira, oregano and rosemary at 1 µL/mL and 5 µL/mL for each E.O.As a matter of fact, their effects on the OTA production and the growth of *A. carbonarius* S402 cultures were evaluated, after four days at 28 °C on a Synthetic Grape Medium (SGM). Results showed that *A. carbonarius* growth was reduced up to 100%, when cultured with the E.Os of cinnamon, taramira, and oregano at both concentrations and the thyme at 5 µL/mL. As for the other six E.Os, their effect on *A. carbonarius* growth was insignificant, but highly important on the OTA production. Interestingly, the fennel E.O at 5 µL/mL reduced the OTA production up to 88.9% compared to the control, with only 13.8% of fungal growth reduction. We further investigated the effect of these E.Os on the expression levels of the genes responsible for the OTA biosynthesis (*acOTApks* and *acOTAnrps* along with the *acpks* gene) as well as the two regulatory genes *laeA* and *vea*, using the quantitative Reverse Transcription-Polymerase Chain Reaction (qRT-PCR) method. The results revealed that these six E.Os reduced the expression of the five studied genes, where the *ackps* was downregulated by 99.2% (the highest downregulation in this study) with 5 µL/mL of fennel E.O.As for the *acOTApks*, *acOTAnrps*, *veA* and *laeA*, their reduction levels ranged between 10% and 96% depending on the nature of the E.O and its concentration in the medium.

## 1. Introduction

Mycotoxins are secondary metabolites produced by a wide range of filamentous fungi which have adverse effects on humans, animals and crops that result in illnesses and economic losses [1]. The most known mycotoxins are the aflatoxins, patulin, citrinin, fumonisin B1, zearalenone and the Ochratoxin A (OTA). In fact, OTA is produced by different species of *Aspergillus* and *Penicillium* such as *A. ochraceus*, *A. carbonarius*, *P. verrucosum* and *P. nordicum.* However, *A. carbonarius* is considered to be the main producer of OTA on coffee and grapes [2]. OTA has nephrotoxic, hepatotoxic, teratogenic, and immunotoxic effects *on* several animal species [3] and was classified by the International Agency for Research on Cancer (IARC) in 1993, as a possible human carcinogen (group 2B) [4]. Additionally, it was associated with the acute renal failure in humans [5] and liver cancer in animals after a long-term exposure with the molecule [6].

Chemical and physical treatments are considered to be inefficient at removing the OTA from grapes and wine without altering their organoleptic properties [7]. Hence, the awareness of the hazardous effects of chemical preservatives has led the scientific community to search for naturally occurring molecules that are able to reduce OTA production without altering the fungal growth, thus preventing its replacement by other undesired mycotoxigenic fungi.

Essential oils (E.Os), herbs and spices antimicrobial properties have been acknowledged and used since ancient times for therapy and food preservation. Thus many studies were carried out on their ability to reduce mycelial growth and/or mycotoxins production by different mycotoxigenic fungi. Soliman and Badeaa [8] found that the E.Os of the thyme, cinnamon, marigold, spearmint, basil and quyssum had inhibitory activities against the growth of *A. flavus*, *A. parasiticus*, *A. ochraceus* and *Fusarium verticillioides.* Similar results were obtained by Basílico and Basílico [9], who found that the mint and oregano E.Os also had a complete inhibitory effect on the growth of *A. ochraceus*.

To date, the biosynthetic pathway of OTA in *A. carbonarius* has not yet been completely elucidated and only few genes have been discovered. However, it was confirmed by several studies that the OTA biosynthesis pathway contains a polykétide synthase (PKS) and a non ribosomal peptide synthase (NRPS) family. A study conducted by Gallo et al. [10] identified and characterized an *acpks* gene in *A. carbonarius* that encodes a conserved ketosynthase and acyl transferase domains, and found that the OTA production strongly depended on the expression levels of the *acpks* gene, proving the implication of this gene in the OTA biosynthesis. Those same authors have identified the *acOTApks* gene that has a different function from *acpks*, the previously described gene. *AcOTApks* encodes an AcOTApks protein that belongs to the (HR)-PKS family, and contains a putative methyltransferase domain likely responsible for the addition of the methyl group to the OTA polyketide structure [11]. Another characterized OTA biosynthesis gene in *A. carbonarius*is the *acOTAnrps* which was proven to be essential for OTA, OTα and OTB biosynthesis and is located about 900 nt upstream of a *pks* gene [12]. Furthermore, the OTA biosynthesis in *A. carbonarius* is regulated by two known genes: *laeA* and *veA*. Their deletion caused a drastic decrease in the OTA production and a dowregulation in the expression of the *nrps* gene [13].

Until now, the effect of E.Os on the expression of the genes responsible for OTA production in *A. carbonarius* is not yet evaluated. Therefore, the aim of this study is to evaluate the effect of 10 different E.Os (anise, rosemary, thyme, oregano, fennel, cardamom, chamomile, cinnamon, taramira and celery) at concentrations of 1 µL/mL and 5 µL/mL, on the OTA produced by *A. carbonarius* (the main contaminant of grapes), and their influence on the expression levels of *acpks*, *acOTApks*, *acOTAnrps*, *laeA* and *veA* genes involved in the OTA biosynthesis, in order to choose the E.O(s) whose effects are only limited to OTA reduction without affecting the growth of *A. carbonarius*.

## 2. Results

### 2.1. Essential Oils Effect on Growth of A. carbonarius S402 and Its OTA Production

The effect of the ten E.Os used in this study at 1 µL/mL and 5 µL/mL, on the *A. carbonarius* dry weight, its radial growth and OTA production are shown in Table 1.

Our results showed that the radial growth, dry weight and OTA inhibition levels strongly depend on the nature of the E.O and its concentration (Table 1). The E.O of taramira, oregano and cinnamon (1 µL/mL and 5 µL/mL) were able to reduce *A. carbonarius* S402 growth up to 100%, with no visible growth on the SGM medium; therefore, no detectable OTA concentration in the culture medium was found after HPLC (High Performance Liquid Chromatography) analysis. Although the E.O of thyme at 5 µL/mL completely blocked the growth of *A. carbonarius* S402, it only decreased 48.71% of the fungal growth at 1 µL/mL.

E.Os of the fennel, cardamom, chamomile, rosemary, anise and the celery at 1 µL/mL and 5 µL/mL, showed a slight effect on the dry weight and radial growth of *A. carbonarius* S402 but a significant impact on the OTA production. A reduction of 88.9% in OTA was reached when 5 µL/mL of fennel E.O was added to the SGM medium, while it only reduced 13% of the fungal growth. Additionally, this E.O at 1 µL/mL was able to reduce the OTA to 86.6%, with no significant impact on the radial growth (33.3%) and the dry weight (11%). Cardamom, chamomile, rosemary, anise and celery E.Os at 1 µL/mL, also had an important effect in OTA reduction that reached 74.2%, 67.5,% 53.7%, 76.6%, and 68.5% respectively, with growth reduction that ranged between 0% and 11% (cardamom and celery (0%), anise (5%), chamomile and rosemary (11%)). OTA reduction was higher, when the E.O concentrations were at 5 µL/mL reaching 83.5% for cardamom E.O with only 13% of dry weight reduction. The OTA reduction percentages were almost the same for the E.Os of chamomile (79.1%), rosemary (78.3%), and celery (77.1%), with a growth reduction of 13%, 8% and 5%, respectively. Lastly, the E.O of anise reduced the fungal growth to 16% with 83.9% of OTA concentration reduction.

### 2.2. Microscopic Morphology Evaluation

**Figure 1:**
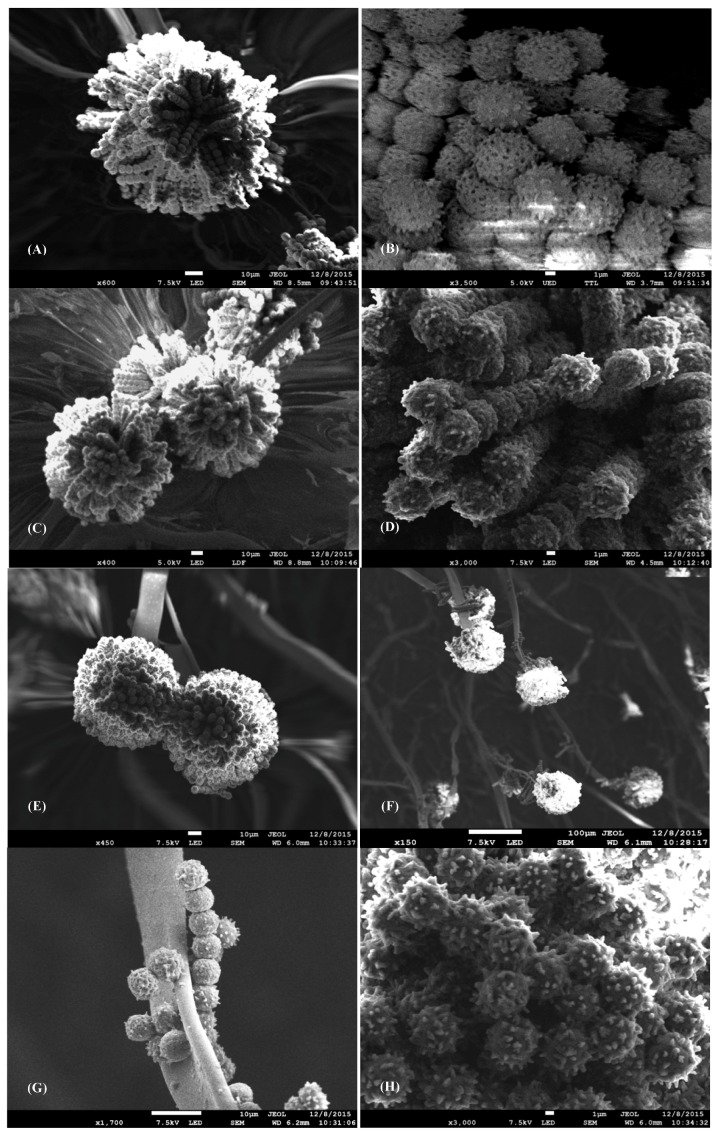
Scanning electron micrographs of the *Aspergillus carbonarius* S402, cultured on Synthetic Grape Medium (SGM) at 28 °C for four days: (**A**) *A. carbonarius* S402 conidial head; (**B**) spores of *A. carbonarius*; (**C**) *A. carbonarius* conidial head cultured with 1 µL/mL fennel oil; (**D**) spores of *A. carbonarius* cultured with 1 µL/mL fennel oil; (**E**,**F**) *A. carbonarius* conidial head cultured with 5 µL/mL fennel oil; (**G**) conidiophores of *A. carbonarius* cultured with 5 µL/mL fennel oil with abnormally placed spores; and (**H**) spores of *A. carbonarius* cultured with 5 µL/mL fennel oil.

Scanning Electron Microscopy (SEM) of *A. carbonarius* S402 treated with 1 µL/mL and 5 µL/mL of fennel E.O compared to untreated samples showed that the application of the fennel E.O at 1 µL/mL did not affect the size of the conidial head. For instance, the conidial head of the untreated samples measured 107 μm (Figure 1A), similar to the treated sample with 1 µL/mL (102 μm) (Figure 1C). When the SGM medium was supplemented with 5 µL/mL of fennel E.O, the results were different: the conidial head was smaller and more compact than those in control condition, and measured 84 μm (Figure 1E), i.e., 21.4% lesser than the control.

As for the spore concentration, a non-significant variation was observed between the untreated *A. carbonarius* and the cultures treated with 1 µL/mL and 5 µL/mL of fennel E.O. In fact, the results showed that the untreated samples had a concentration of 17 × 16^6^ spores/mL, whereas the cultures treated with 1 µL/mL and 5 µL/mL of fennel E.O had a concentrations of 15.8 × 10^6^ spores/mL and 16.1 × 10^6^ spores/mL respectively.

### 2.3. Analysis of Genes Expression Involved in OTA Biosynthesis by A. carbonarius

To better understand the OTA reduction by the E.Os that did not affect the fungal growth, the expressions of two known biosynthetic genes involved in the OTA biosynthesis (*acOTApks* and *acOTAnrps*) as well as the *acpks* gene and the two regulatory genes (*veA* and *laeA*) were evaluated by qRT-PCR.

The expression levels of the target genes were normalized by the expression of the reference gene (β-*tubulin* was chosen as reference gene, as shown in the Supplementary Material, Figure S1) and were expressed relative to the control. Table 2 shows the relative expression values of the target genes of *A. carbonarius* S402 cultured with the six E.Os, along with the OTA reduction.

Results showed that expression level of the target genes in the presence of 1 µL/mL of E.Os varied depending on the nature of the E.O. In fact the E.O of the chamomile, was able to significantly downregulate all the target genes. Others, such as cardamom, rosemary, anise, fennel and celery E.Os, were only able to affect specific genes but had no effect on the others. As a matter of fact, the *acpks* was significantly downregulated when *A. carbonarius* was cultured with the six E.Os at 1 µL/mL. Notably, its expression was reduced by 96% by the E.O of the fennel, 50% and 90% by the celery and rosemary E.Os respectively, accompanied by an OTA reduction that reached 86.9%, 68.8% and 53.7% respectively for each E.O. As for the cardamom, chamomile and anise E.Os, they were able to equally reduce this gene expression by 80% and had approximately the same effect on OTA production, as they reduced it to 74.2% and 76.9% by cardamom and anise E.O, respectively, and to 67.5% by the chamomile E.O.

As for the *acOTApks*, its expression was not downregulated by neither of the six E.Os. In fact, E.O of the rosemary had no significant effect on this gene’s expression while the other tested E.Os reduced the *acOTApks* by 99%, 86%, 88%, 81% and 76% for the fennel, cardamom, chamomile, anise and celery E.Os, respectively. While *acOTAnrps* expression was equally reduced by the E.Os of cardamom, chamomile and rosemary by 80%, its expression was not affected by the fennel, celery and anise E.Os.

Regarding the regulatory genes *veA* and *laeA*, they were both not affected by the E.Os of the anise and celery. The *veA* expression was reduced by approximately 90% with the E.Os of the fennel and rosemary, along with 55% and 63% by the E.Os of cardamom and chamomile respectively. As for the *laeA*, it was downregulated by 92%, 71% and 80% with the E.Os of the fennel, chamomile and rosemary respectively. However, the E.O of the cardamom had no significant effect on the *laeA* expression.

At 5 µL/mL, the six E.Os were able to significantly reduce the expressions of the five studied genes. For instance, the *acpks* was highly downregulated by the E.O of the fennel, reaching 99.2% of reduction, followed by the chamomile, rosemary, cardamom and celery E.Os with 98%, 95%, 90%, 85% and 70% reduction, respectively.

As for the *acOTApks*, its expression was reduced up to 99.9% with the fennel E.O, the highest gene downregulation in this study. Moreover, this E.O decreased the OTA production up to 88.6% yet reduced the fungal growth by only 13.6%. The E.Os of the cardamom, rosemary and chamomile also had major effects on the expression of the *acOTApks*. In fact they reduced its expression by 98%, 96% and 95%, respectively, coupled with an OTA reduction by 83.5%, 78.3% and 79.1%, respectively, for each E.O. In addition, celery and anise E.Os downregulated the *acOTApks* by 81%, and 86% respectively as well as the OTA production by 77.1% and 83.9% for each E.O respectively. As for the E.O of fennel, rosemary and anise, they were able to reduce the *acOTAnrps* expression by 98% for both fennel and rosemary, and 99% for anise. Regarding the rest of the E.Os, the cardamom, celery and chamomile, they also reduced this gene expression by 84%, 82% and 52% respectively.

Finally the *veA* and *laeA* expressions were nearly the same when *A. carbonarius* was cultured with the E.Os of the fennel (93% and 94%), cardamom (71% and 70%), chamomile (94% and 96%), rosemary (95% and 91%), anise (87% and 80%) and lastly celery (88% and 80%), respectively, for each gene.

## 3. Discussion

Until now, studies have shown that the E.Os can reduce OTA production by reducing fungal growth. Nevertheless, few were able to prove that OTA could also be inhibited without significantly reducing the fungal growth. To that purpose, this work was dedicated to finding an E.O capable of reducing the OTA produced by *A. carbonarius*, without significantly altering its growth, thus preserving the natural balance of the microbial ecosystem and mostly preventing the occurrence of other mycotoxigenic fung.

However, few works were able to prove that the reduction of OTA production could be associated with the downregulation of the expression of genes responsible for the OTA biosynthesis in *A. carbonarius*. In this study, we investigated the effects of ten E.Os on OTA production by *A. carbonarius* S402 and we found that four of these E.Os, taramira, oregano, cinnamon and thyme, completely blocked OTA production by preventing the growth of *A. carbonarius* S402 cultures. These findings are consistent with other studies found in the literature, which tested the effect of some E.Os on the growth of other mycotoxigenic fungi. In fact, Hua et al. [14] found that *A. ochraceus* fungal growth was completely blocked by using the E.O of cinnamon, a natural and synthetic cinnamaldehyde with a concentration of 500 µL/L for the three E.Os. Moreover, the antifungal activities of the cinnamon was studied over the years and proven to be effective against mould growth [8,15,16,17]. These results regarding the E.O of the cinnamon are consistent with our results, stating that the growth of *A. carbonarius* S402 was completely blocked with the use of this E.O. The oregano E.O also had a great antifungal activity, preventing the growth of *A. niger*, *F. oxysporum* and *Penicillium spp.* in YES broth, at a concentration of 0.1 mL/mL [18]. Additionally, the carvacrol and thymol were identified as the main components of the oregano E.O, responsible for its antifungal activities [19,20,21]. Similarly, the E.O of the thyme also showed an antifungal effect on the growth of *F. verticillioides*, *A. parasiticus*, *A. flavus* and *A. parasiticus* due to the presence of the thymol, thyme’s main component [22,23,24].

However, the E.O of anise, fennel, cardamom and chamomile did not show a significant antifungal activity against *A. carbonarius* at the concentrations used in this study. The E.O of anise at 1500 µL/L, when used on *A. ochraceus* on a Malt Extract Agar medium (MEA) [14] did not show a significant effect on the growth of the fungus, which similar to our results but contradict those of Soliman and Badeaa [8] who found that at 500 ppm, this E.O blocked the growth of *A. flavus*, *A. ochraceus*, *A. parasiticus* and *F. verticillioides* on PDA medium (Potato Dextrose Agar) after 5–7 days of culture. Furthermore, these same authors found that the E.O of the fennel at 3000 ppm blocked the growth of the four tested fungi, also contradicting with our results, stating that, even at 5 µL/mL (0.5%), this E.O only reduced *A. carbonarius* S402 growth by 13.8% compared to a control. A study conducted by Bansod and Rai [25] showed that the MIC of the fennel E.O against the growth of *A. fumigatus* and *A. niger* was at 2% of this E.O, which means that in order to block the growth of these two fungi, a concentration 4 times higher than ours was needed. The reasons behind these differences could be numerous; first of all, they may be associated with the fact that in our study, we did not extract the E.Os from the plants but instead we bought them from the market; consequently, the extraction methods may vary and alter the antifungal activity of the E.Os. Furthermore, different species of fungi do not have similar spores density or similar growth rates (*A. carbonarius* has a negligible ratio of (spores dry weight)/(biomass dry weight) on DG18 agar medium compared to *A. flavus* and *A. ochraceus*, but it has a significantly higher biomass density than the other two fungi due to its larger conidial size compared to *A. flavus* and *A. ochraceus*, respectively)[26], consequently causing the E.Os to perform differently on different fungal species. Lastly, these contradictions may be associated with the different types of media used in both studies along with the incubation period.

According to Baydar et al. [27] the antifungal properties of E.Os can be attributed to the presence of phenolic compounds such as eugenol, anethole, carvacol, precursors *r*-cimene and *g*-terpinolene and isomers of thymol. Furthermore, they have low molecular weight and lipophilic properties that allows them to easily penetrate cell membrane [28] causing irreversible cell wall damage and cellular organelle as well as affecting the pH homeostasis and equilibrium of inorganic ions [29,30,31]. Another study on the mode of action of E.Os on fungal growth suggested that *A. conyzoides* E.O was able to cross the plasma membrane of *A. flavus* and interact with the membrane structures of cytoplasmic organelles thus preventing the fungal growth [32].

Regarding the expression levels of the genes involved in the OTA biosynthesis pathway, in presence of E.O, few studies were found in the literature on this topic. Nevertheless, Murthy et al. [33] noticed that the reduction of OTA production was not correlated with the growth reduction of *A. ochraceus* after inoculating the medium with increasing doses of Ajowan Ethanolic Extract (AEE). At 50 and 150 mL/g of AEE, inhibition of OTA production was higher than the reduction of fungal growth. Similarly, Hua, Xing, Selvaraj, Wang, Zhao, Zhou, Liu and Liu [14] also noticed a decreased growth of *A. ochraceus* by 75–150 mg/mL of citral E.O, but the OTA production was completely blocked, therefore suggesting that the reduction was not correlated with the growth reduction, but rather to the suppression of transcription of OTA biosynthesis genes. In fact, they found that the transcription of the *pks* gene (responsible for the synthesis of the polyketide dihydroisocoumarin and involved in the first steps of the OTA biosynthetic pathway) was completely inhibited by 75–150 mg/mL of citral E.O. Likewise; a study conducted by Caceres et al. [34] on the reduction of aflatoxin B1 (AFB1) produced by *A. flavus* showed that all the genes (except for *aflT*) of the AFB1 cluster were drastically downregulated when *A. flavus* was cultured with 0.5 mM of eugenol, resulting an extreme AFB1 reduction with no significant impact on the fungal growth. In the same manner, our study revealed that six of the tested E.Os (anise, fennel, cardamom, chamomile, celery and rosemary) reduced significantly the OTA from the medium without equally reducing the fungal growth. These results suggest that this OTA reduction was not associated with the growth reduction but rather with the repression of certain biosynthetic genes. Fennel, cardamom, chamomile, rosemary, anise and celery E.Os downregulated the *acpks*, *acOTApks*, *acOTAnrps*, *laeA* and *veA* genes, involved in the synthesis of the appropriate enzymes involved in the OTA biosynthesis. A similar study revealed that the expression of the *acOTApks* gene in *A. carbonarius* was also reduced by the addition of 0.065 mg/mL of the hydroxycynnamic acids such as *p*-coumaric and ferulic acids [35]. Our findings suggest that the E.Os used in this study have different ways to reduce OTA from the medium without affecting the fungal growth. Some were able to downregulate the regulatory genes (*laeA* and *veA*) such as the fennel, chamomile and rosemary E.Os at 1 µL/mL and consequently reducing the expression of the genes responsible for the OTA biosynthesis (*acpks, acOTApks* and *acOTAnrps*). Others, such as the anise and celery E.Os at 1 µL/mL did not have significant effect on the expressions of *laeA* and *veA*, but they were able to downregulate directly the *acpks, acOTApks* and *acOTAnrps* genes, suggesting that different types of E.Os has different mode of action on the OTA biosynthesis genes. Similarly, a study was conducted on *F. culmorum* cultured with 0.5 mM of ferulic acid showed that this compound reduced the trichotecene type B production by reducing up to 5.4 times the expression levels of several *tri* genes involved in the biosynthesis pathway [36]. Furthermore, AFB1 production was reduced by adding the caffeic acid in culture with *A. flavus*, resulting in the downregulation of the aflatoxins biosynthesis genes in *A. flavus*, reducing 6.6 fold the *aflD*, 7.1 *aflM*, 9.1 *aflP* and 1.5 *aflS* gene without affecting the fungal growth [37]. Moreover, another study conducted by Yoshinari et al. [38] found that AFG1 production in *A. parasiticus* was reduced by using E.O of the chamomile that inhibited the cytochrome P450 synthesis (CYPA), enzyme implicated in the AFG1 production. Additionally, due to the similarity between the CYPA and TRI4 (an enzyme responsible for trichothecenes early biosynthesis), the E.O of chamomile was also able to decrease the 3-acetyldeoxynivalenol (3-ADON) production in *F. graminearum* by reducing the TRI4 enzyme.

Regarding the microscopic aspects of *A. carbonarius* cultured with 1 µL/mL and 5 µL/mL of fennel E.O, the morphology of the conidial head and the spores remained unchanged, contrary to other results found in the literature. For instance, Hua, Xing, Selvaraj, Wang, Zhao, Zhou, Liu and Liu [14] found that the treatment of *A. ochraceus* with different concentrations of natural cinnamaldehyde, citral and eugenol E.Os showed alteration in the morphology of the hyphæ that appeared collapsed and abnormal, correlated with an OTA reduction. Moreover, the E.O of *Cinnamomum zeylanicum* caused an interesting inhibition of spore germination in *A. flavus*, *A fumigatus* and *A. niger* also causing alterations in the hyphal development, a loss in pigmentation and a lack of sporulation [39]. Another similar study conducted by Sharma and Tripathi [40] showed that the E.O of *Citrus sinensis* at 0.5 μg/mL caused a distortion of the *A. niger* mycelium, squashed and flattened the conidiophores bearing damaged conidial head. In our case, the E.O of the fennel used in this study highly reduced OTA production without altering the growth of *A. carbonarius* S402 or the microscopic aspects of the fungus.

Our findings, along with the data collected from previous studies, indicate that the use of the E.Os on food and crops could be one of the solutions to reduce the OTA from culture media produced by *A. carbonarius*. Some of these E.Os, including anise, celery, cardamom, chamomile, rosemary and fennel, were able to reduce the OTA by repressing the expressions of the genes involved in the OTA biosynthesis without particularly affecting the fungal growth; thus not altering the natural microbial balance.

## 4. Conclusions

The inhibitory effects of ten E.Os on the OTA production of *A. carbonarius* S402 and their effect on the expression of the genes involved in OTA biosynthesis were evaluated in this study. The E.Os of cinnamon, thyme, oregano and taramira showed an antifungal activity and prevented the growth of *A. carbonarius* S402 cultured on SGM medium. Nevertheless, the objective of our study was to find one or several E.O that has no significant effect on *A. carbonarius* growth, but rather on OTA production. For this aim, we found that the E.O of fennel at 5 µL/mL was the most effective E.O in terms of downregulating the OTA biosynthesis and regulatory genes of *A. carbonarius*, in correlation with a drastic reduction in the OTA production. In addition, this E.O did not alter the morphology of the fungus’ conidial heads or the sporulation after four days of culture on SGM medium. Similarly, the E.O of the chamomile, cardamom, celery, rosemary and the anise did not show any antifungal activities and thus did not have a significant effect on the growth of the fungus, but only reduced the OTA production by reducing the genes expressions responsible for this production.

## 5. Materials and Methods

### 5.1. Essential Oils

Ten E.Os were chosen in order to evaluate their influence on the growth of *A. carbonarius* and on OTA production: rosemary (*Rosmarinu sofficinalis*), anise (*Pimpinella anisum*), chamomile (*Chamaemelum nobile*), fennel (*Foeniculum vulgare*), thyme (*Thymus vulgaris*), oregano (*Origanum vulgare*), taramira (*Eruca sativ*), cinnamon (*Cinnamomum verum*), cardamom (*Elettariacardamomum*) and celery (*Apium graveolens*). These E.Os were purchased from the local Lebanese market (Tyr, Lebanon), and were used with no additional treatment.

### 5.2. Strain and Culture Conditions

*A. carbonarius* strain S402 was provided by the LGC-UMR 5503 (Chemical Engineering Laboratories) located in the Superior National School of Agronomy, in Toulouse France. This strain was sub-cultured in Czapec-Yeast-Agar medium (CYA) as described by El Khoury, Rizk, Lteif, Azouri, Delia and Lebrihi [2] for 4 days at 28 °C. A spore suspension was prepared by adding 8 mL of a sterile Tween 80 (0.005%) solution and scrapping the surface of the cultures with a sterile Pasteur Pipette (Chase Scientific Glass, Inc, Rokwood, TN, USA). The spore count was evaluated by using a Neubauer haemocytometer (Superior, Marienfeld, Lauda-Konigshofen, Germany), the concentration was adjusted to 10^6^ spore/mL and spore suspension was then kept at 4 °C for further use. The culture was then conducted on a Synthetic Grape Medium (SGM), prepared following the instructions described by Bejaoui et al. [41].

Each E.O (at 1 µL/mL and 5 µL/mL) was mixed to 20 mL of the SGM medium before pouring the mix into Petri dishes. In order to compare and evaluate the effect of the E.Os on *A. carbonarius* growth and on OTA production, a control culture was prepared without adding any E.O to the SGM medium. A sterilized transparent cellophane film was placed on the surface of the medium, and then 10^6^ spores of *A. carbonarius* were inoculated at the center of the film. The growth of *A. carbonarius* was estimated by the radial growth (cm) and the dry weight (g), both after 4 days of culture. *A. carbonarius* dry weight was evaluated by weighing the mycelium after its desiccation (placing the transparent film carrying the culture at 100 °C for 24 h). As for the radial growth, diameters measurements of *A. carbonarius* cultures were taken after 4 days of incubation at 28 °C.

All assays were carried out in triplicates for each condition.

### 5.3. Scanning Electron Microscopy (SEM) for Morphologic Study and Spores Count

The fennel E.O at 1 µL/mL and 5 µL/mL was chosen among the ten E.Os to observe its effect on the spores and conidia morphology of *A. carbonarius* in comparison with the control on SGM medium. This E.O was chosen in order to investigate its effect on the fungal sporulation since it had the greatest effect on the OTA production without altering significantly the growth of *A. carbonarius*. After 4 days of culture at 28 °C, the fungus was desiccated at 75 °C for 24 h, prepared for the SEM by coating the samples with 40 nm of gold under vacuum and then viewed with the MEB-FEG (Field Emission Gun); model JSM 7100F (JOEL, Freising, Germany). The spore concentration was assessed using a Neubauer haemocytometer (Superior, Marienfeld, Lauda-Konigshofen, Germany), after taking 4 agar plugs from each condition and mixed with 5 mL of distilled water. The tubes were then shaken vigorously using vortex mixer for 3 min. Samples were made in triplicates and the mean number of spores was considered.

### 5.4. OTA Extraction and HPLC Analysis

After the incubation period, 3 agar plugs (0.5 cm diameter) were extracted from each SGM medium, placed in 3 mL microtubes and weighed. One milliliter of HPLC grade methanol was added, and the mix was incubated and shaken for 60 min at room temperature. After centrifugation at 13,000 r.p.m (round per minute), the aqueous phase was separated from the debris and diluted with 20 mL phosphate buffer. OTA was then purified using Ochraprep immunoaffinity columns (R-Biopharm, Glasgow, Scotland) by injecting the mix into the columns using a syringe. OTA was eluted by adding 1.5 mL of methanol/acetic acid (98:2, *v/v*) followed by 1.5 mL of distilled water. After filtering with 0.4 μm filters (Sartorius stedim, Biotech), the OTA extract was stored at 4 °C before quantification.

OTA quantification was done with a Water Alliance HPLC system using an Utisphere ODB column, C18 (150 × 4.6 mm, 5 µm, 120 Å) (Interchim, Montluçon, France) at 30 °C. A 30 min isocratic flow was delivered at 49% of eluent A: acidified water (0.2% of acetic acid) and 51% of eluent B: acetonitrile. A flow rate of 1 mL/min was used and 10 µL of extract were injected into the apparatus. OTA was detected by a fluorescent detector at 333/440 nm excitation/emission wavelengths. Peak identity was confirmed by analyzing absorption spectrum with a diode array detector coupled to the system. OTA concentrations were calculated based on a standard calibration curve.

### 5.5. RNA Extraction, cDNA Synthesis and qRT-PCR

For genes expressions analysis, culture conditions were the same as those described in Section 5.2. After 4 days at 28 °C, the cellophane film was separated from the surface of the SGM medium and the mycelium was then carefully cut out from the cellophane, ground in liquid nitrogen and stored at −80 °C. Total RNA was extracted from the frozen mycelium using the RNeasy kit (Qiagen, Düsseldorf, Germany) following the manufacturer protocol and then treated with DNase I Amplification Grade (Sigma-Aldrich, St. Louis, MO, USA). The quality and quantity of the total RNA was assessed using the Experion RNA analysis kit (version 3.20, 2015, BioRad, Marnes-la-Coquette, France). The single strand cDNA was synthesized from 1 μg of total RNA using the Advantage RT-for-PCR kit (Clontech, Mountain View, CA, USA) following the manufacturer protocol.

Gene specific primers used are listed in Table 3. The reactions were held using CFX96 Touch Real time PCR detection system Bio-Rad (version 3.0, 2012) using Sso Advanced Universal Sybr Green Supermix (Bio-Rad, Marnes-la-Coquette, France) to evaluate the cDNA amplification, by starting with 100 ng/µL of freshly synthesized cDNA following the manufacturer protocol for the mix preparation and the amplification cycles, with a Tm of 58 °C. The choice of suitable reference genes was conducted by choosing six candidate genes (β-*tubulin, cox5, actin, 18S, calmodulin and gpdA*) used in previous studies, and their stability was tested in the conditions used in our study using the qRT-PCR (version 3.0, 2012, Bio-Rad, Marnes-la-Coquette, France) method and the geNorm software (version 3.0, 2014, Biogazelle, Ghent, Belgium). Their expression stability was assessed with qbase^+^ biogazelle software (version 3.0, 2014, Biogazelle, Ghent, Belgium) by calculating their geNorm *M* value (*M*) and their coefficient of variation on the normalized relative quantities (*CV*). These values can then be compared against empirically determined thresholds for acceptable stability [42]. The most stable gene is the one with the lowest *M* value and with a *CV* value that does not exceed 0.15 (*M* value of < 0.5 and *CV* value of < 0.15).

The PCR efficiencies for each primer pair were determined by a serial cDNA dilution experiments, and were calculated from the slopes of the curves given by the CFX96 Touch Real time PCR detection system software (Bio-Rad).

### 5.6. Statistical Analysis

Data regarding the dry weight, radial growth, OTA concentrations and genes expressions were analyzed through one-way analysis of variance (ANOVA) and paired *t*-test using GraphPad (version 6.07 for windows, 2015, GraphPad Software Inc., La Jolla, CA, USA) prism 6 software.

## Figures and Tables

**Table 1:** Dry weight (g) and diameter (cm) of *Aspergillus carbonarius* S402, the OTA concentration (ng/mL*g) after four days of culture at 28 °C on Synthetic Grape Medium(SGM) supplemented with the ten essential oils at 1 µL/mL and 5 µL/mL, compared to a control.

Essential Oils	Dry Weight (g)		Radial Growth (cm)		OTA Concentration (ng/mL*g of Dry Weight)	
Control	0.36 ± 0.05 ^a^		5.1 ± 0.01 ^c^		50.58 ± 0.79	
	5 µL/mL	5 µL/mL	1 µL/mL	5 µL/mL	1 µL/mL	5 µL/mL
Anise	0.38 ± 0.01 ^a^	0.3 ± 0.01 ^b^	4.5 ± 0.01 ^c^	3.5 ± 0.05 ^c,d^	16.66 ± 0.55 *	10.36 ± 0.27 *
Cardamom	0.36 ± 0.005 ^a^	0.31 ± 0.005 ^b^	4.5 ± 0.01 ^c^	4.4 ± 1 ^c^	13.15 ± 0.20 *	8.37 ± 0.15 *
Celery	0.36 ± 0.05 ^a^	0.34 ± 0.05 ^a^	4.1 ± 0.05 ^c^	3.8 ± 0.00 ^d^	16.43 ± 0.24 *	15.60 ± 0.24 *
Chamomile	0.36 ± 0.005 ^a^	0.31 ± 0.005 ^b^	5.1 ± 0.05 ^c^	4.5 ± 0.01 ^c^	16.54 ± 0.26 *	10.66 ± 0.19 *
Cinnamon	No growth	No growth	N.D	N.D	N.D	N.D
Fennel	0.32 ± 0.05 ^b^	0.31 ± 0.005 ^b^	4.1 ± 0.05 ^c^	3.4 ± 0.01 ^c^	6.8 ± 0.12 *	5.6 ± 0.10 *
Oregano	No growth	No growth	N.D	N.D	N.D	N.D
Rosemary	0.36 ± 0.01 ^a^	0.33 ± 0.001 ^a^	4.5 ± 0.00 ^c^	4.5 ± 0.00 ^c^	23.62 ± 0.65 *	11.03 ± 2.80 *
Taramira	No growth	No growth	N.D	N.D	N.D	N.D
Thyme	0.2 ± 0.01 *	No growth	2.1 ± 0.01 *	N.D	3.56 ± 0.17 *	N.D

The Mean of the dry weight (g) and the growth inhibition (%) ± the standard deviation of the triplicates are represented in this table. Statistical differences are indicated as: * = significant difference (*p* < 0.01). Data with the same letters are not significantly different (*p* < 0.05). N.D: Not Detectable.

**Table 2:** Normalized relative Ochratoxin A (OTA) gene expression in *Aspergillus carbonarius* S402 observed after 4 days of culture in presence of six different E.Os at 1 µL/mL and 5 µL/mL, associated with the percentage of OTA reduction levels.

Essential Oils	Fennel				Cardamom				Chamomile			
	1 µL/mL		5 µL/mL		1 µL/mL		5 µL/mL		1 µL/mL		5 µL/mL	
Genes	Relative expression	% of OTA reduction	Relative expression	% of OTA reduction	Relative expression	% of OTA reduction	Relative expression	% of OTA reduction	Relative expression	% of OTA reduction	Relative expression	% of OTA reduction
*acpks*	0.04 *	86.90%	0.008 *	88.60%	0.24 *	74.20%	0.15 *	83.50%	0.2 *	67.50%	0.02 *	79.10%
*acOTApks*	0.009 *		0.001 *		0.14 *		0.02 *		0.12 *		0.05 *	
*acOTAnrps*	0.7		0.02 *		0.2 *		0.18 *		0.2 *		0.16 *	
*veA*	0.09 *		0.07 *		0.45 *		0.29 *		0.37 *		0.06 *	
*laeA*	0.08 *		0.06 *		0.9		0.3 *		0.29 *		0.04 *	
**Essential Oils**	**Rosemary**				**Anise**				**Celery**			
	**1 µL/mL**		**5 µL/mL**		**1 µL/mL**		**5 µL/mL**		**1 µL/mL**		**5 µL/mL**	
Genes	Relative expression	% of OTA reduction	Relative expression	% of OTA reduction	Relative expression	% of OTA reduction	Relative expression	% of OTA reduction	Relative expression	% of OTA reduction	% of OTA reduction	Relative expression
*acpks*	0.1 *	53.70%	0.05 *	78.30%	0.2 *	76.90%	0.1 *	83.90%	0.5 *	68.50%	0.3 *	77.10%
*acOTApks*	0.9		0.04 *		0.19 *		0.14 *		0.24 *		0.19 *	
*acOTAnrps*	0.2 *		0.02 *		0.8		0.01 *		0.7		0.48 *	
*veA*	0.1 *		0.05 *		0.9		0.13 *		0.7		0.12 *	
*laeA*	0.2 *		0.09 *		0.7		0.2 *		0.8		0.2 *	

Normalized gene expression of the following genes: *acpks, acOTApks, acOTAnrps, veA* and *laeA* along with the OTA reduction in the Synthetic Grape Medium (SGM) after four days of culture at 28 °C. Statistical analysis was made using the GraphPad prism 6 software (version 6.07 for windows, 2015, GraphPad Software Inc., La Jolla, CA, USA). Data with (*) = significant difference (*p* < 0.01).

**Table 3:** List of primers used in this study.

Primer Name	Primer Sequence (5’→3’)	References	Efficiency
*acpks-F*	GAGTCTGACCATCGACACGG	[10]	110.0%
*acpks-R*	GGCGACTGTGACACATCCAT		
*acOTApks-F*	CGTGTCCGATACTGTCTGTGA	[11]	102%
*acOTApks-R*	GCATGGAGTCCTCAAGAACC		
*acOTAnrps-F*	ATCCCCGGAATATTGGCACC	[12]	82.6%
*acOTAnrps-R*	CCTTCGATCAAGAGCTCCCC		
*laeA-F*	CACCTATACAACCTCCGAACC	[13]	104.7%
*laeA-R*	GGTTCGGCCAACCGACGACGC		
*veA-F*	TCCCGGTTCTCACAGGCGTA	[13]	101.3%
*veA-R*	GCTGTCCTTGGTCTCCTCGTA		
*β-tubulin-F*	CGCATGAACGTCTACTTCAACG	[43]	95%
*β-tubulin-R*	AGTTGTTACCAGCACCGGA		
*calmodulin-F*	CCAGATCACCACCAAGGAGC	[44]	105%
*calmodulin-R*	GTTATCGCGGTCGAAGACCT		
*18S-F*	GCAAATTACCCAATCCCGAC	[44]	98%
*18S-R*	GAATTGCCGCGGCTGCTG		
*cox5-F*	CCCTGTTCTACGTCATTCACTTGTT	[45]	87%
*cox5-R*	TCTTCTCGGCCTTGGCATACTC		
*actin1-F*	TTGACAATGGTTCGGGTATGTG	[45]	82%
*actin1-R*	TTGACAATGGTTCGGGTATGTG		
*gpdA-F*	ACGGCAAGCTCACTGGTATGT	[45]	110%
*gpdA-R*	CAGCCTTGATGGTCTTCTTGATG		

## Supplementary Materials

The following is available online at www.mdpi.com/2072-6651/8/8/242/s1., Figure S1 Genorm *M* value of the six candidate reference genes: β-*tubulin, calmodulin, cytochrome c oxydase subunit V* (*cox5*), *18S, actin* (*actin1*) and *glyceraldehydes 3-phosphate dehydrogenase* (*gdpA*). The most stable gene is the one with the lowest *M* value.

## Abbreviations

The following abbreviations are used in this manuscript:

*A. carbonarius*	*Aspergillus carbonarius*
qRT-PCR	Quantitative Reverse Transcription-Polymerase Chain Reaction
OTA	Ochratoxin A
IARC	International Agency for Research on Cancer
E.O	Essential oil
SGM	Synthetic Grape Medium
HPLC	High Performance Liquid Chromatography
mM	Milliomolar
N.D	Not detectable
r.p.m	Round per minute
