# Supplementary material for: Essential Oils Modulate Gene Expression and Ochratoxin A Production in Aspergillus carbonarius
Source: Toxins (Basel). 2016 Aug 19;8(8):242. doi: 10.3390/toxins8080242 (PMC4999858; doi:10.3390/toxins8080242)
Supplement: Supplementary file 1 [file toxins-08-00242-s001.pdf]

Supplementary Materials: Essential Oils Modulate Gene Expression and Ochratoxin A Production in *Aspergillus carbonarius*

Rachelle El Khoury, Ali Atoui, Carol Verheecke, Richard Maroun, Andre El Khoury and Florence Mathieu

1. Choice of a Suitable Reference Gene for the Gene Expression Study

In order to appropriately choose a reference gene for this study, *β-tubulin*, *calmodulin*, *cytochrome c oxydase subunit V* (*cox5*), *18S*, *actin* (*actin1*) and *glyceraldehydes 3-phosphate dehydrogenase* (*gdpA*)genes were tested using the GeNorm software (version 3.0, 2014, Biogazelle, Ghent, Belgium). Results showed that *β-tubulin* (geNorm M = 0.2) and the *actin* (geNorm M = 0.24) genes displayed a higher stability regardless the cultures conditions, and can be successfully used in our study as reference genes (Figure S1).For this reason, *β-tubulin* was selected as the reference gene.

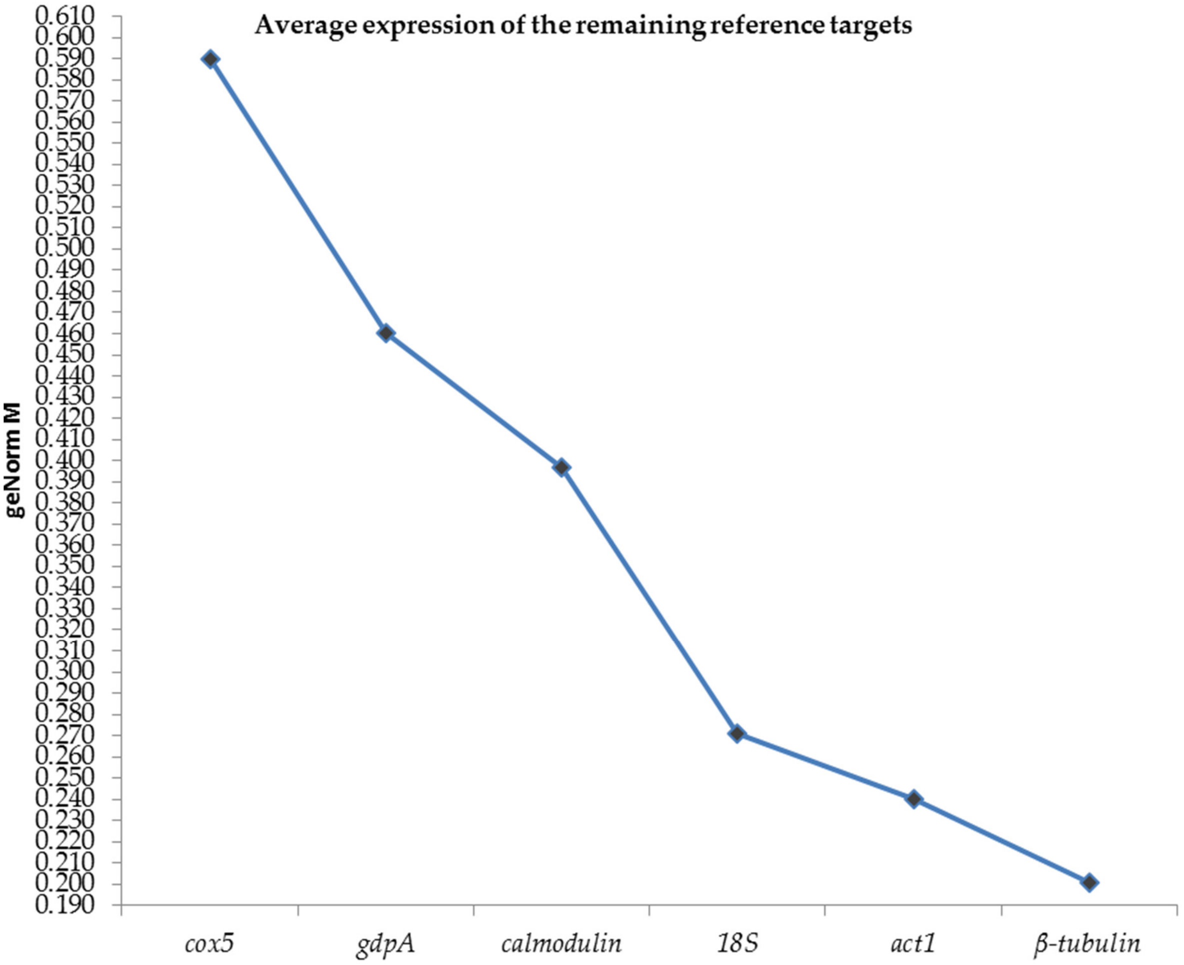

Figure S1. Genorm M value of the six candidate reference genes: *β-tubulin*, *calmodulin*, *cytochrome c oxydase subunit V* (cox5), *18S*, *actin* (*actin1*) and *glyceroldehydes 3-phosphate dehydrogenase* (*gdpA*). The most stable gene is the one with the lowest M value.
